# Missed opportunities in antenatal care for improving the health of pregnant women and newborns in Geita district, Northwest Tanzania

**DOI:** 10.1186/s12884-018-2014-8

**Published:** 2018-10-05

**Authors:** Eveline Thobias Konje, Moke Tito Nyambita Magoma, Jennifer Hatfield, Susan Kuhn, Reginald S. Sauve, Deborah Margret Dewey

**Affiliations:** 1 Department of Biostatistics & Epidemiology, School of Public Health, Catholic University of Health and Allied Sciences, P.O. box 1464 Bugando Area, Mwanza, Tanzania; 2 Department of Community Health Sciences, Cumming School of Medicine, University of Calgary, 3280 Hospital Drive, NW, Calgary, AB Canada; 3 Options Tanzania Ltd 76 Ali Hassan, Mwinyi Road, P.O. Box 65350, Dar es Salaam, Tanzania; 4 Department of Paediatrics, University of Calgary, 2888 Shaganappi Tr. NW, Calgary, AB Canada; 5 Owerko Centre at the Alberta Children’s Hospital Research Institute, Cumming School of Medicine, University of Calgary, 2500 University Dr. NW, Calgary, AB Canada

**Keywords:** Antenatal care, Missed opportunity, Tanzania, Sequential explanatory mixed method

## Abstract

**Background:**

Despite the significant benefits of early detection and management of pregnancy related complications during antenatal care (ANC) visits, not all pregnant women in Tanzania initiate ANC in a timely manner. The primary objectives of this research study in rural communities of Geita district, Northwest Tanzania were: 1) to conduct a population-based study that examined the utilization and availability of ANC services; and 2) to explore the challenges faced by women who visited ANC clinics and barriers to utilization of ANC among pregnant women.

**Methods:**

A sequential explanatory mixed method design was utilized. Household surveys that examined antenatal service utilization and availability were conducted in 11 randomly selected wards in Geita district. One thousand, seven hundred and nineteen pregnant women in their 3rd trimester participated in household surveys. It was followed by focus group discussions with community health workers and pregnant women that examined challenges and barriers to ANC.

**Results:**

Of the pregnant women who participated, 86.74% attended an ANC clinic at least once; 3.62% initiated ANC in the first trimester; 13.26% had not initiated ANC when they were interviewed in their 3rd trimester. Of the women who had attended ANC at least once, the majority (82.96%) had been checked for HIV status, less than a half (48.36%) were checked for hemoglobin level, and only a minority had been screened for syphilis (6.51%). Among women offered laboratory testing, the prevalence of HIV was 3.88%, syphilis, 18.57%, and anemia, 54.09%. In terms of other preventive measures, 91.01% received a tetanus toxoid vaccination, 76.32%, antimalarial drugs, 65.13%, antihelminthic drugs, and 76.12%, iron supplements at least once. Significant challenges identified by women who visited ANC clinics included lack of male partner involvement, informal regulations imposed by health care providers, perceived poor quality of care, and health care system related factors. Socio-cultural beliefs, fear of HIV testing, poverty and distance from health clinics were reported as barriers to early ANC utilization.

**Conclusion:**

Access to effective ANC remains a challenge among women in Geita district. Notably, most women initiated ANC late and early initiation did not guarantee care that could contribute to better pregnancy outcomes.

**Electronic supplementary material:**

The online version of this article (10.1186/s12884-018-2014-8) contains supplementary material, which is available to authorized users.

## Background

To reduce preventable maternal and newborn mortality and morbidity, the provision of quality antenatal care (ANC), accessible obstetrics care, and life-saving interventions are essential services [1–5]. In 2015, approximately 303,000 maternal deaths occurred worldwide with sub-Saharan Africa accounting for 66% of all maternal deaths [6]. Although some developing countries have achieved up to a 75% reduction in the maternal mortality ratio (MMR) since 1990, Tanzania has made little progress over the past 25 years [2, 6]. In 1990, the MMR was 529 maternal deaths per 100,000 live births and in 2015/16 it was 545 maternal deaths per 100,000 live births [7]. Most developing countries have achieved a reduction in mortality among under five children; however, the proportion of neonatal deaths and stillborn babies remains unacceptably high [2, 8]. Globally, it was estimated in 2015 that there were 18.4 stillbirths per 1000 births with rural populations accounting for 60% of all stillborn babies [8]. Tanzania ranked ninth in the world among the ten countries with the highest number of stillbirths in 2015 with an estimated 47,000 stillbirths [8]. Most of these stillbirths occurred during labor and delivery and were considered to be due to preventable or manageable health conditions [8, 9].

The sustainable development goal 3.1, emphasizes reducing the MMR to less than 220 per 100, 000 live births for countries with an MMR above the average global level [10]. To end preventable causes of maternal deaths due to pregnancy related complications, scaling up of existing interventions during pregnancy and delivery is crucial [5]. The main causes of maternal deaths are hemorrhage, hypertensive disorder, and infection [11]. Newborn deaths and stillbirths are mainly due to asphyxia, prematurity, and infections [8, 9] and most are preventable with existing evidence-based interventions either directly or indirectly during pregnancy, labor, delivery, and post delivery [1, 3, 5].

Safe motherhood initiative programs recognize the significance of the provision of quality care from preconception to the postnatal period for women and newborn babies. They consist of four pillars, namely, family planning, ANC, clean and safe delivery, and essential obstetric care services [3]. ANC is recognized and emphasized due to its influence on the wellbeing of pregnant women and their unborn babies [1, 3, 5]. In 2002, Tanzania adopted the Focused ANC (FANC) model recommended by the World Health Organisation (WHO). It is still utilized although the WHO introduced a new ANC model in 2016 [1, 12]. The FANC model emphasizes individualized care for all pregnant women as any pregnancy could face complications. It suggests a minimum of 4 visits for uncomplicated pregnancies with the initial visit occurring before 16 weeks gestation, the second visit between 20 and 24 weeks, a third visit between 28 and 32 weeks, and a fourth visit at 36 weeks [13].

Although universal coverage of ANC services is reported globally and at the country level [2, 7, 10, 14], in developing countries, many pregnant women fail to benefit from comprehensive ANC due to factors such as late initiation and the poor quality of ANC services [2, 14–17]. Early initiation during the first trimester and quality ANC over the pregnancy period has been recognized as improving pregnancy outcomes and increasing newborn survival [18, 19]. However, in developing countries, only 25% of pregnant women initiated ANC before 14 weeks gestation and 48% of pregnant women did not complete 4 ANC visits [2, 15]. Several factors have been associated with late initiation including: older age, higher parity/multiparity, lower education level, hidden costs, lack of male support, pregnancy-related cultural beliefs, unplanned pregnancy, and health system related issues such as shortages of supplies and drugs [14, 20–24]. However, most of the studies that have reported on the timing and frequency of ANC utilization were retrospective in nature and included women who had a live birth in the 2–5 years prior to the conduct of the study. The results of these studies could be influenced by survival and recall bias, as the information obtained was from women who survived their pregnancy and were able to provide a self report [14, 20–22, 25, 26]. In addition, these studies were health facility-based, and as a result may suffer from selection bias as attendees were likely to be women with better health care seeking behavior than non-attendees, particularly in rural low income settings, leading to an over estimation of early initiation and utilization of ANC during pregnancy [20, 21, 25].

In Tanzania, early initiation of ANC (i.e., attending antenatal care in the first trimester) is reported to be 24%, and only 51% of women had more than 4 antenatal care visits in 2015/16 [7]. Northwest Tanzania is a region with a low rate of utilization of ANC services despite the existing high fertility rate in this region [7]. The primary objectives of this research study in rural communities of Geita district, Northwest Tanzania were: 1) to conduct a population-based study that examined the utilization and availability of ANC services, and 2) to explore the challenges faced by women who visited ANC clinics and barriers to utilization of ANC among pregnant women.

## Methods

### Study setting

The study was conducted at Geita district, one of the six districts in Geita region Northwest Tanzania. The district has 35 wards with a district hospital, 5 health centers, and 38 dispensaries. Eleven wards (31%) were randomly selected for inclusion in the study, namely: Lwamgasa, Nyaruyeye, Bukoli, Nyarugusu, Nyakamwaga, Butundwe, Chigunga, Nyamiluluma, Bukondo, Nzera, and Lwenzera. Random selection was accomplished by first alphabetically ordering the wards and numbering them from 1 to 35. Then a random numbers table was used to select eleven wards. Based on the Tanzania Demographic and Health Survey (TDHS) 2015/16, Geita performed poorly on components of ANC and more than a half (52.3%) of pregnant women delivered at home [7].

### Study design

This study is part of a large cohort study that is investigating utilization of maternal and child health services, pregnancy outcomes, birth-related complications, and maternal and child mortality and morbidity up to 4 months postpartum. The study utilized a sequential explanatory mixed method approach. This method has two phases. The first phase is characterized by the collection and analysis of quantitative data. The second phase involves the collection and analysis of qualitative data. This method uses the qualitative findings to further explain and interpret the findings of the quantitative data and was selected as it allowed exploring in detail the challenges pregnant women faced when accessing ANC services and the barriers to utilization of ANC services.

### Quantitative data collection process

A household survey using a cross sectional design involving pregnant women in the third trimester was conducted between September 2016 and August 2017. Based on 2015 total deliveries for the entire district (36,101), we assumed that approximately 10,000 (28%) pregnant women would be residing in the study area with at least 20% (2000) of them being in their third trimester of pregnancy. The study reached 1805 (90%) of the expected pregnant women in the selected wards, of whom 1719 participated in the survey. Of those who were contacted but not included in our final sample, 81 were not residents of a study area, two had had miscarriages, one woman was in the second trimester of her pregnancy based on her last menstruation period, and two women refused to participate because they were not feeling well.

The household survey consisted of a face-to-face interview. During the conduct of the household survey interview, only pregnant women in their 3rd trimester who voluntarily consented were invited to participate in the study. In the situation that a pregnant woman was not at home during the time the household survey was conducted, that household was re-visited up to three times to see if the woman was interested in participating. Village leaders, community health workers, and traditional birth attendants assisted in the identification of households with pregnant women. Trained research assistants who were registered nurses, and intern medical doctors conducted the household survey. The research assistants were not health care providers at health facilities in the study areas (they were from college/university and were not employed as health care providers); hence, interview bias was expected to be minimal. The principal investigator (EK) supervised the conduct of the survey.

For the household survey, a structured pretested questionnaire was utilized to capture baseline information including socio-demographic characteristics, gestational age, parity, gravidity, obstetric history, immunization status, intermittent preventive treatement status, deworming status, and the use of iron and folate supplements. The questionnaire also captured information on birth preparedness, an anticipated place for delivery, social support after delivery, money saving for any emergency during pregnancy, and the purchase baby’s items, which are included as part of the health education provided through ANC services (seeAdditional file 1). For women who had attended ANC clinics, responses to the household survey questions were crosschecked with their antenatal cards, which are provided to pregnant women who attend ANC clinics. During the household survey, the pregnant women’s blood pressure was checked, weight and height were measured, an identification card for follow-up purposes was provided, and counselling regarding the utilization of ANC services was provided. The household surveys were conducted in the homes of the pregnant women unless otherwise specified by the participant. In those rare cases (only four women), the interview was scheduled at a convenient location identified by the participant, usually at their farm or at a friend’s house.

### Qualitative data collection process

A case study was conducted to explore in depth barriers to utilization of antenatal care services among women in the study area. Women and community health workers from six wards namely Lwenzera, Nzera, Nyarugusu, Bukoli, Lwamgasa, and Bukondo were invited to participate in focus group discussions. Using the results from the analyses of the household survey data, study wards were selected based on the criterion of having a significant number of women who initiated ANC late or who never attended ANC. Community health workers (CHWs) were also invited to participate because of the nature of their work in bridging the community with the health care system. Hence, participants were purposively selected for the focus group discussions (FGDs). FDGs provided the women with a safe environment in which they could present their views and opinions and provided the participants with the opportunity to see that the challenges and barriers that they experienced were similar those of other women. The FGDs also promoted open discussion and sometimes disagreement, and allowed us to observe the group dynamics. Finally, FGDs allowed us to observe whether there was consensus of group members on the challenges faced by women who visited ANC clinics and the barriers to utilization of ANC among pregnant women.

We conducted six FGDs with women who had recently delivered babies, many of whom had not utilized ANC and delivery services. Thirty-five women from the selected wards participated in the FGDs with an average of five to six women in each group. Six focus groups were also conducted with CHWs, one in each of the six wards. On average, five to six CHWs, both male and female participated from each ward making 32 CHWs. A semi-structured interview guide was used to facilitate discussion among participants. The key issues explored were: 1) What do you know about maternal and child health services available to you during pregnancy or to pregnant women? 2) What has been your experience with ANC services in your community? and 3) Why do some pregnant women not utilize the ANC services available in their communities or are late in utilizing these services?

The research team conducted the FDGs in the Swahili language and where necessary in the local language (Sukuma). Members of the research team who were fluent in Sukuma assisted with translation during discussions. None of the research team members involved in conducting the FGDs provided directed medical care to the participants through the local community health centers. Voice recordings were transcribed, translated into English, and back translated into Swahili to ensure content consistency. Field notes were taken by EK and some of the FGDs were supervised by DD. For the purposes of confidentiality, privacy, and friendly environment, FGDs typically occurred at the village leaders’ offices or at primary schools. However, in some cases, (two focus groups), they took place at the local health clinic. Each FGD took approximately one hour and thirty minutes.

### Data management and analysis

ANC service utilization was the outcome of interest. Three levels were examined, namely no attendance at ANC services, attendance within the first trimester, and attendance in the second or third trimester. Epi-Data version 3.1 software was used for data entry with the double entry system feature to reduce data entry errors. This feature allows double entry of the same questionnaire data by two different clerks. During dataset validation, inconsistencies were resolved by reviewing the original questionnaires and editing accordingly. Cleaned data were exported and analyzed using STATA version 13 [27]. The 95% confidence intervals and *p* values reported were based on a 5% level of statistical significance. Chi-squared tests were used to examine associations between categorical variables.

Qualitative data were transcribed and translated into English by two RAs fluent in Swahili and English and cross-checked by EK for discrepancies. Thematic analysis was conducted by EK and reviewed by DD. It involved familiarization with data, identification of the main themes, indexing, charting, mapping, and interpretation. Line by line coding was done manually and identified themes were compared with written field notes for convergence or divergence of ideas. The identified themes were used to gain a deeper understanding of the quantitative results. The data source triangulation was done by having group discussions with community health workers and women in order to confirm the perceived challenges and barriers. The contiguous approach was adapted for data integration at the interpretation and reporting level.

## Results

### Household survey participant characteristics

Of the 1805 pregnant women visited at their homes, 1719 were eligible for this study. The average age of the participants was 25.7 years (see Table 1). Almost all of the participants (94.71%) were married and the majority of the women reported having a primary school education (68.59%); however, a considerable proportion (23.61%) reported no formal schooling.

**Table 1:** Socio-demographic characteristics of 1719 pregnant women in rural Geita by ANC attendance

Characteristic	Overall		Women attended ANC at least once (*N* = 1491)		Women not attended ANC (*N* = 228)	
	Mean(SD)	n (%)	n (%)	Mean (SD)	n (%)	Mean (SD)
Maternal age (in years)	25.73 (6.60)			25.51 (6.52)		27.28 (6.96)
Maternal height (in cm)	155.66 (6.90)			155.77 (6.73)		154.89 (7.92)
Maternal weight (in Kg)	59.49 (8.39)			59.71 (8.38)		57.97 (8.31)
Marital status						
Currently single		91 (5.29)	83 (5.57)		8 (3.51)	
Currently married		1628 (94.71)	1408 (96.43)		220 (96.49)	
Education level						
No formal education		406 (23.61)	347 (23.27)		59 (25.88)	
Primary		1179 (68.59)	1024 (68.68)		155 (67.98)	
Secondary & above		134 (7.80)	120 (8.05)		14 (6.14)	

### Household survey

#### Antenatal services utilization and availability

Overall, antenatal attendance with at least one visit was 86.74% (1491/1719); however, a considerable proportion of participants 13.26% (228/1719) had not initiated ANC at the time of the household survey. Of the 1491 pregnant women who visited an antenatal clinic at least once, only 3.62% (54/1491; 95% CI 2.67–4.57) initiated ANC at the first trimester while the rest of the participants initiated ANC either in the 2nd or 3rd trimester. Further, although more than three quarters of participants attended antenatal clinics, provision of laboratory and preventive services were not common to all pregnant women. As per the FANC model, pregnant women should receive services related to disease prevention, health promotion, detection and treatment of existing disease, and information on developing a birth preparedness plan. Laboratory testing was limited to 82.96% women for HIV, 48.36% women for hemoglobin level (Hb in g/dL), and 6.51% for syphilis (see Table 2). The prevalence of existing conditions based on the records from antenatal cards was 3.88% for HIV infection, 54.09% for anemia, and 18.57% for syphilis infection. Regarding preventive measures, 91.01% of participants received a tetanus toxoid vaccination, 76.32%, antimalarial drugs, 65.13%, antihelminthic drugs, and 76.12%, iron supplements at least once.

**Table 2:** Antenatal care services received and prevalence of screened conditions among 1491 women

Characteristic	Services received at clinics	Prevalence	95% CI
	n (%)	n (%)	
HIV Status			
-Total Screened	1237 (82.96)		
-Tested Positive		48 (3.88)	2.80–4.95
-Tested Negative		1189 (96.12)	
Hemoglobin level			
-Total screened	721 (48.36)		
-Hb level < 10.9 g/dL		390 (54.09)	50.45–57.74
-Hb level > =10.9 g/dL		331 (45.91)	
Syphilis status			
-Total Screened	97 (6.51)		
- Tested Positive		18 (18.57)	10.68–26.43
- Tested Negative		79 (81.43)	
Iron supplements	1135 (76.12)		
Antihelminthic drugs			
- None	520 (34.87)		
- One Dose	475 (31.86)		
- Two Doses	496 (33.27)		
Intermittent preventive treatment			
- None	353 (23.68)		
- One Dose	518 (34.74)		
-Two Doses	620 (41.58)		
Tetanus toxoid vaccine			
- None	134 (8.99)		
- One Dose	229 (15.36)		
-Two or more doses	1128 (75.65)		

During the household survey the women were asked about their intentions to deliver at the health facility; plans for transport to the health facility, and saving money for possible emergencies during pregnancy or delivery. They were also asked if they had bought any items for their baby (i.e., basin, clothes, soap), and any social support after the delivery of the baby (i.e., their birth preparedness plan). More than half (54.33%) of the participants indicated that they did not intend to deliver at a health facility. The majority (71.5%) had no plan for transport during labour and 64.39% had not purchased baby items. However, half of the participants had saved money (49.74%) for an emergency during pregnancy or delivery. Significant differences (*p* value < 0.05) were observed between women who attended ANC clinics at least once and those who did not attend ANC on all components of birth preparedness except social support. ANC clinic attendees were more likely to plan for transport, save money, buy items for their baby, and indicate that they intended to deliver at a health facility compared to those who had not attended an ANC clinic (Table 3).

**Table 3:** Birth preparedness planning among 1719 pregnant women in rural Geita district

Characteristic		Overall	Women who attended ANC at least once *N* = 1491	Women who had not attended ANC *N* = 228	Difference in proportions *P* value (chi^2^ test)
		n (%)	n (%)	n (%)	
Health facility delivery	Yes	785 (45.67)	730 (48.96)	52 (24.12)	< 0.05
Transport preparation	Yes	490 (28.50)	448 (30.05)	42 (18.42)	< 0.05
Saving money	Yes	855 (49.74)	783 (52.52)	72 (31.58)	< 0.05
Purchase baby items	Yes	574 (33.39)	531 (35.61)	43 (18.86)	< 0.05
Social support	Yes	1438 (83.65)	1254 (84.10)	184 (80.70)	0.20

#### General characteristics of participants in FGDs

The women who participated in six FGDs (*n* = 35) were multiparous and had attended ANC clinics at least once in previous pregnancies or the current pregnancy. Of the 32 CHWs who participated in the FGDs, all had at least a year of experience working as a CHW and over half (53%) were male. In terms of the dynamics of the FGDs, the women and the CHWs were very open and forthcoming in their responses to the questions asked by research team members, most women and CHWs contributed to the discussion and participation by all participants was encouraged by the researchers.

#### Perceived barriers for antenatal care services

This section discusses the perceived barriers that women identified as hindering their utilization of ANC services. It focuses on the issues that women perceived to hinder their utilization of available ANC services. Focus group discussions with women and CHWs were used to identify themes related to perceived barriers to utilization of ANC services in rural Geita district. Women indicated that they do not initiate ANC at all due to the following reasons: poverty, fear of HIV testing, and socio-cultural beliefs (Table 4).

Poverty may influence the health care seeking behavior of pregnant women negatively. Many women have no source of income in the family; therefore, any cost related to health care is a financial burden for the entire family. Fares for transport to ANC clinics, a maternity dress, or other hidden costs can act as a barrier for some women in the rural communities.

*Having no income in the family, and [the] health facility being far may lead to women not attending ANC services at all considering the family does not have even a bicycle. Female participant 1*

*It could be poverty, since nurses here emphasize clean clothes and proper dress such as a maternity dress when a woman wants to attend clinic. If you don’t have a maternity dress you may not go. Female participant 5*

Participants also identified men’s lack of interest and their unwillingness to participate during pregnancy, which resulted in some women not attending ANC clinics. For HIV prevention of mother-to-child transmission, couples are required to go together to the first antenatal visit. This practice aims at providing counselling and HIV testing to both partners. However, some health providers deny ANC services to pregnant women who attended without their male partners. Fear of HIV testing by both female and male partners was also perceived to be a barrier to ANC for couples that were unwilling to participate in HIV testing together.

*Some women stay at home throughout their pregnancy period because men are not ready to accompany them to the clinic. It is a “must to go” with your husband on the first visit. Female participant 1*

*Yes, men not escorting their women is a main problem I can say. When a pregnant woman attends clinic for the first time, the health provider must asks where is your husband, and if you don’t have your male partner the possibility of being seen in the clinic is very small and you may end up being scolded. To avoid being harassed, pregnant women may not attend the ANC clinic. Male CHW 1*

In the rural settings, parents or in-laws make decisions for couples who live with them. Decisions related to health care seeking may depend upon the attitudes of parents/in-laws towards ANC services and their experiences with the health care system. Men who escort their wives are considered weak in this male dominant society. Participants indicated that parents/in-laws might hinder pregnant women from attending ANC clinics and it becomes a barrier when the couple depends on their parents/in-laws financially.

*It is a habit based on parents’ experiences. Men do think if my mother did not attend clinic at all or attended only once and gave birth without any problem why bother now. It is common with couples that stay with their parents. If your wife mentions about escorting her to the clinic, your father may say in our times, we did not go with women to their services, why do you want to do it now. This is being weak, your mother did not go and all went well. Then you think no need for my wife to attend clinic. Male CHW 5*

*Sometimes, a few men may prevent women from attending ANC arguing, do women who miss out ANC face any problems during delivery? But there are those who attended and still experienced complications. So you will stay at home, all will be well. With this response, some women decide not to attend at all. Female participant 6*

*For those families that stay with their in-laws, since the mother-in-law did not attend clinic, a pregnant woman will find it difficult to attend ANC clinic. Since her mother-in-law will discourage her saying, “We did not go to ANC clinic in those years why do you want to go there?” It becomes more difficult if a man depends on his parent financially. Female CHW 6*

### Challenges faced by women in visiting ANC clinics

This section highlights the challenges faced by pregnant women who attended an ANC clinic at least once. These challenges included poverty, perceived poor quality of ANC services, and lack of male involvement that hindered pregnant women’s timely utilization of ANC services (Table 4).

Participants stated clearly that poverty delayed pregnant women from accessing ANC services in a timely manner. Due to the distance to health facilities and lack of transportation, many pregnant women decided to wait until their third trimester to initiate visits to an ANC clinic. Early ANC initiation meant spending a lot of money on transport, and many women had no source of ongoing income and lacked a bicycle or motorcycle for transport. Factor such as distance may act as both a barrier as well as a challenge to the utilization of ANC services. For some women distance to the ANC clinic could delay their attendance until the third trimester. As a result, they would only make a single visit during their pregnancy for a general check-up.

*Due to distance and other issues, instead of visiting to the clinic every month a woman opts to attend only once to avoid the frequency of going to the clinic every month by attending in the last months of the pregnancy from seventh month. Female CHW 1*

*Sometimes you may feel weak and lazy to walk every month, remember we cannot afford paying for boda boda because we don’t have money. Then you decide to wait till you approach the 8*^*th*^ *or 9*^*th*^ *month so that you have one visit to get an ANC card*. *Female participant 3*

*Long distance for some of the villages discourages most of the pregnant women to attend ANC clinics early. Some villages are far away from dispensaries, approximately more than 7 kilometers, making it difficult for pregnant women to walk that long distance. This is worse when the household does not have a bicycle or a motorcycle or money for fare. Even if they know the importance of ANC services, still they may not attend early. Male CHW 1*

Women appeared to have some knowledge of the benefits of initiating early ANC services; however, perceived poor quality of ANC services in this community discouraged timely initiation of care. Pregnant women are supposed to receive comprehensive ANC when attending clinics, but in these communities, the absence of health care providers and shortages of supplies and drugs were identified as common challenges.

*Most of the services such as antimalarial drugs, iron tablets, mosquito nets are not always available, and laboratory supplies are on and off because our clinics serve a large population, which is not proportional to the available supplies and drugs. Female CHW 1*

*When you go for ANC they send you back several times because health providers are not there or have gone for seminars. You may go to clinic several times with no luck of receiving any ANC services till you get tired and give up. You may miss the services for several visits because the health providers have gone for seminars or training. Female participant 5*

*This practice of sending back pregnant women without services has been common in our dispensary and it discourages pregnant women. For example, recently there were no ANC services for the entire week because of the shortage of health providers. There was only one health provider attending only out patients and other emergency care services. Male CHW 6*

Lack of male involvement and participation during pregnancy was also a challenge for women who attended ANC clinics in the rural communities. Participants noted that the lack of men’s involvement or interest was associated with cultural beliefs, the influence of in-laws, and the environment at the health facilities, which was not male friendly. HIV testing is crucial for both partners; however, and both partners need to agree to HIV testing after proper counselling and health education. In this community, HIV testing was associated with fear by the women as the laboratory test was done in the presence of the male partner. Furthermore, the existing health facilities provided no privacy for the couple during the counselling or the conduct of HIV testing.

*Actually, most women in this community go late for antenatal services because of the fear of HIV testing while some men refuse to escort their women for fear of HIV testing. Female participant 4*

*It is really challenging in this community because of the high rates of HIV infection. The habit of HIV testing among men is not there. Escorting women to the clinic requires men to be ready for HIV testing and results. They think how I can go there when I am not ready to take an HIV test. It is better I just send my wife and I will know if I am safe or not through my wife’s HIV result. They believe if a woman is HIV negative they are also negative so there is no need of going there. Male CHW 4*

*We do not have a friendly environment. For example, with our health facility there is no infrastructure for a reproductive and child health unit. Currently, all patients who come for TB and HIV drugs, out patients, and pregnant women and under-five children are gathered in one place. There is no privacy and friendly environment for the couple. Male CHW 3*

Some cultural beliefs in relation to pregnancy were perceived as causing delays in visiting ANC clinics. For example, in the first trimester, women do not disclose their “invisible” pregnancy to people including health providers for the fear of being witched. In addition, there were misconceptions about use of hematinics as many women thought that iron supplements prolonged the period of pregnancy, tended to exaggerate morning sickness symptoms, and sometimes even cause adverse outcomes. To avoid prolonged use of hematinics, they delay the initiation of ANC care.

*We wait for a visible pregnancy for us to start clinic. You cannot start attending clinic with an invisible pregnancy; our fellow women may scold you when we go to fetch water. We do not mention to everyone that you are pregnant. We do this because we fear being witched since you cannot know who your enemy is. Female participant 6*

*You may visit the household with the aim of identifying a pregnant woman. Nobody in the family would dare to disclose that. To your surprise a few months late, you may meet the same woman with a newborn baby. Misconceptions on haematinics are also a challenge because pregnant women think when you use iron or folate you may experience abortion, fetus progress delay, and annoying side effects. Male CHW 5*

*Misconception regarding use of iron tablet/syrup exists among women thinking that when you use these drugs you delay the growth of the pregnancy while others complain about the side effects of the drugs such as nausea. So they prefer to visit clinic late to avoid continued use of these drugs. Female CHW 4*

### How women cope with existing ANC requirements

In these communities, some women circumvent ANC requirements during the first antenatal visit. Since attendance of male partners and HIV testing are mandatory during the first visit, participants reported visiting a clinic away from their ward, and carrying a letter from village leaders confirming that their husband was not present. In addition, some pregnant women paid for men who were willing to escort them and take the HIV test with them, a so-called “husband for hire” Table 4.

*Husband for hire do exist in our villages. These are boda boda men. When a woman gets a boda boda to take her to the health facility, she also requests if the man is willing to escort her for HIV testing just to pretend being a husband. Female CHW 6*

*Since men will not accept escorting their wives to the antenatal clinic for fear of HIV testing, women decide to go to the village leaders for the letter explaining that the husband for this woman is not present in the village and may be working outside the district. They get a letter because health providers insist if you come without your husband you will not get any service. Female CHW 5*

*Your husband must be there when you go to the clinic for the first visit. This is a big challenge for women. Therefore, for those who cannot get their husband to escort them* [they] *may go to a clinic far from this ward because nobody knows their husband there. The boda boda driver usually can pretend to be your husband and you pay him something small. Female participant 1*

**Table 4:** Identified themes and subthemes from FGDs

Key issues	Themes	Sub themes
Perceived barriers to utilization of ANC services	Poverty	• Health facility was far from home and pregnant women feeling tied to walking long distances
		• Women or family not having income to afford transport
		• Having no fare for hiring a bicycle or paying for a boda boda
		• Not having a maternity dress
	Fear of HIV testing	• Pregnant women’s fear of HIV testing
		• Male partner fear of HIV testing results
		• Misconception regarding HIV testing
		• Self stigmization arising from HIV results
		• Infidelity among men/risk behaviors in fishing community and mining community
	Socio-cultural beliefs	• Men’s refusal to escort women/men feel no need to be involved
		• Normal practices or habits because no history of pregnancy related complications
		• Parental influence especially among those couples who live with their parents
Challenges faced by women when utilizing the ANC services	Lack of male involvement	• Unfriendly male environment
		• Men not willing to escort women
		• Attended ANC clinic but not provided services as male partner did not attend
	Perceived poor quality of care	• Services not available because supplies and/or drugs are out of stock
		• No services available most of the times
		• Long waiting times for services
		• Frequent shortages of health providers
	Informal regulation	• HIV testing is no longer voluntarily
		• Male partner must be there during the first ANC visit
		• Not receiving HIV testing since a man is not with you
Coping strategies for existing ANC requirements	Men for hire	• Husband for hire do exist in our villages
		• Men will not accept escorting their wives
		• Boda boda men are willing to accompany women if they are provided with a little money for escorting you
	Letter from village leaders	• To avoid being scolded and to obtain services you get a letter from village leader to present to the CHWs
		• If your partner refuses to escort you state that he is away
	Health facility	• Attend a health facility that is far away from your home
		• Where health providers do not know you or your husband

## Discussion

Antenatal care is one of the pillars in the Safe Motherhood Initiative for promoting and improving maternal and child health through interventions such as health promotion, treatment of existing diseases, early detection and management of pregnancy-related complications, and disease prevention [3]. Initiating ANC in the first trimester provides opportunities for timely optimal care and treatment of existing conditions [3, 5, 13]. Our findings revealed that the timely attendance at the ANC clinic in the first trimester of pregnancy was low with more than three quarters of participants first attending either in the second or third trimester. This is a significant missed opportunity for improving maternal and child health in Geita district, Northwest Tanzania. The extremely low attendance (3.62%) of women in Geita district, Northwest Tanzania in the first trimester is not consistent with the levels of attendance in Tanzania (24%) and globally (58.6%) [7, 15]. It is important to note that in contrast to previous retrospective studies, this household survey involved pregnant women in their third trimester. Hence, social desirability and recall biases that could have influenced the estimates reported in earlier studies [7, 15] were less likely to bias our results.

We also observed that a considerable proportion of women failed to utilize ANC services fully during their pregnancy due to several reasons including lack of male involvement or lack of men’s interest in ANC, perceived poor quality of care, poverty and socio-cultural beliefs. Other studies have documented similar issues that may hinder utilization of ANC [22, 25, 26, 28]. For example, to achieve reduction and elimination of HIV infection through mother to child transmission, male involvement has been observed to increase women’s adherence to interventions [29]. In low incomes countries, especially in the rural settings, a shortage of skilled health personnel, lack of drugs and supplies, and long waiting times are common challenges encountered in the health facilities [16, 30–33]. Women in the study area reported similar concerns. Further, ANC requirements such as the male partner being present during the first visit for HIV testing, which is emphasized as one strategy for the prevention of maternal to child transmission (PMTCT) of HIV infection, may lead to unintended consequences as documented in this paper. Although the strategy is intended to promote male involvement for positive pregnancy outcomes, it was reported to impede women from initiating ANC in a timely manner or in some cases women may not initiate ANC. Thus, there is a need to revisit such ANC requirements as they may result in unintended harms to maternal and child health.

While ANC improves and promotes maternal and child health, its effectiveness depends in part on the availability and quality of services provided regardless of the antenatal model implemented in the country [3–5, 19, 34, 35]. However, women in the study setting did not receive all the components of ANC services as per national guidelines. Notably, HIV screening was almost universal but far fewer women were screened for syphilis and anaemia, despite evidence for high rates of syphilis sero-prevalence and anaemia. Low coverage for laboratory services has been mentioned in other studies as a public health challenge in most developing countries [16, 30, 31, 36]. In rural settings, laboratory services for measuring Hb level, syphilis, and HIV infections may not be available in public health facilities due to lack of supplies and limited expertise among health care workers to conduct the tests. Further, in private health facilities, these tests may not be free of charge and many women, particularly in rural districts, may not have the funds to pay for these tests. Strengthening laboratory services in primary care facilities is paramount for quality ANC services and prevention of adverse outcomes such as congenital syphilis, stillbirths, prematurity, low birth weight, and perinatal deaths. Concurrently, early initiation of ANC should be emphasized so that women and their unborn babies will reap the full benefits of ANC.

Normative behaviour and traditional beliefs surrounding pregnancy, labour and delivery must also be addressed, as they appear to shape health-seeking behavior and could negatively affect the well-being of pregnant women and their unborn babies. Importantly, the beliefs that the experience of the mother-in-law applies to her son’s wife and that the risk of adverse pregnancy outcomes may not be mitigated by ANC need to be addressed urgently through appropriate channels. In addition, health system factors such as shortages in service providers and important components of ANC (i.e., laboratory tests, vaccines, drugs, supplements), and the demands attached to the prevention of mother to child transmission services such as mandatory partner attendance at ANC initiation, need to be revisited and addressed. ANC clinics are considered “women spaces”, and the existing infrastructure does not provide any privacy for couples. Women also live in a cultural atmosphere with norms and gender roles that shape and influence their health care decision-making. In the African context, women and young married couples may lack autonomy on health-related issues. The male dominant social structure gives men autonomy over their female partners on different aspects of life including health care issues. Thus, parents, in-laws, or men in the community influencing or making decisions for young couples or women is common in developing countries like Tanzania. Previous research has documented the existence of male dominated social structures and male partners playing significant decision making roles in developing countries [37, 38].

The integration of the quantitative and qualitative data assisted us in understanding the challenges and barriers that these women experience in attaining timely ANC by shedding light on community and health system related factors that need to be addressed. There is a need to understand how social structures, culture and beliefs could enhance or hinder utilization of health services. The imposed informal regulation on male partner involvement in the initial ANC visit that has been implemented to reduce the prevention of maternal to child transmission (PMTCT) of HIV needs to be re-examined using a participatory approach that emphasizes community involvement and engagement. As observed in this study, “husband for hire” and village leaders’ written memos used to navigate the ANC requirements imposed on pregnant women, may fail to achieve their intended purpose. If women are checked for HIV infection with their fake male partners, the goal of promoting safer sex, adherence to PMTCT interventions, and HIV status disclosure between partners will not be realized. Hence, this study highlights the *missed opportunities* associated with early initiation of ANC for promoting health seeking behavior and preventing health conditions that directly or indirectly affect maternal and child health in rural settings in Tanzania.

### Study strengths and limitations

The study design accounted for possible biases inherent in previous retrospective studies. In addition, the study was strengthened by triangulation in data collection and the fact that almost all FGD interviews were conducted outside the health facility environment. Finally, a significant strength of this study is that it explored the perspectives of *women who used* ANC services, *women who did not use ANC* services and *health care providers* regarding the challenges and barriers to ANC services in this area of Tanzania. A potential limitation of this study was the use of information/data from the antenatal cards. This could have led to biased estimates of the prevalence of existing conditions such as anemia, syphilis, and HIV. These parameters depend on the quality and completeness of the records of the pregnant women who attended ANC clinics at least once.

## Conclusion and recommendations

The study highlights the low attendance of pregnant women at ANC clinics in the first trimester in Geita district, Northwest Tanzania and the limited antenatal services provided to women who utilized ANC services at least once. The goal of improving and promoting maternal and child health through ANC remains elusive in this rural setting. Importantly, not all components of ANC are available to pregnant women even when they initiate ANC early. The critical shortage of human resources, particularly when an ANC provider is invited to attend training away from the health facility further limits women’s access to timely ANC services. Based on the findings, in-job training of health providers should be well planned to avoid inconvenience and delays in the provision of care. Improving human resources and timely availability of all essential components of ANC, and a friendly environment for male partners will ensure acceptability and quality services in this and similar rural settings. Further, supportive supervision to health workers during the provision of ANC services and training that specifically focuses on providing services to pregnant women and their partners in an open and sensitive manner needs to be implemented to improve the uptake of ANC services in rural communities. HIV screening for prevention of PMTCT should be conducted voluntarily after provision of health education and counselling and health workers need to ensure that they observe all ethics around ANC services. Finally, community male champions need to be identified. These individuals would take a leading role in exploring and promoting male involvement in maternal and child health services in local communities through family visits, community meetings, and cultural and religious gatherings. Such initiatives are essential for improving the health and outcomes of mothers and newborns.

## Additional file

Additional file 1: A structured pretested questionnaire. It provided the quantitative data from household survey on timing and utilization of antenatal care services among pregnant women in Geita district, Northwest Tanzania. (DOCX 25 kb)

## Abbreviations

ANC: Antenatal care
CHW: Community health worker
FANC: Focused antenatal care
FGD: Focused group discussion
HIV: Human immunodeficiency virus
MMR: Maternal Mortality Ratio
PMTCT: Prevention of mother to child transmission
RCH: Reproductive and Child Health
TDHS: Tanzania Demographic and Health Survey
WHO: World Health Organisation
