# Supplementary material for: Missed opportunities in antenatal care for improving the health of pregnant women and newborns in Geita district, Northwest Tanzania
Source: BMC Pregnancy Childbirth. 2018 Oct 5;18:394. doi: 10.1186/s12884-018-2014-8 (PMC6173847; doi:10.1186/s12884-018-2014-8)
Supplement: Supplementary file 1 — A structured pretested questionnaire. It provided the quantitative data from household survey on timing and utilization of antenatal care services among pregnant women in Geita district, Northwest Tanzania. (DOCX 25 kb) [file 12884_2018_2014_MOESM1_ESM.docx]

## C1-A: Household survey data collection tool (Questionnaire English Version)

**Baseline information on maternal, pregnancy, and health services related characteristics**

**Date of a survey: ___/___/____ Initial of Enumerator: |______|**

**Questionnaire no.:|______| Participant Identification no.:|_____|**

**District: ______________ Ward: _________________ Village: ______________**

| **SECTION A: Socio-demographic characteristics of pregnant woman** | | | |
| --- | --- | --- | --- |
| **1.** | | Maternal age in complete years  Record Date of Birth: [dd/mm/yyyy] /____/____/_________/ | \|____\|years |
| **2.** | | Maternal height in centimeters | \|____\|cms |
| **3.** | | Maternal weight in kilogram (Kg) | \|____\|.\|____\|kgs |
| **4.** | | Marital status: **(circle response)**   1. Married 2. Single 3. Divorced/Separated 4. Widower | |
| **5.** | | Highest education level: **(circle response)**  1. Didn’t go to school 2. Primary level 3. Secondary level 4. Tertiary level | |
| **SECTION B: Antenatal care services utilization & birth preparedness** | | | |
| **6.** | Have you attended an antenatal clinic for this pregnancy? **(circle response)**  1. Yes 2. No | | |
| **7.** | Do you have an *ANC card for your index pregnancy? **(circle response)**  1. Yes 2. No  ****ANC card (Antenatal clinic card)[Ask participant if you can see her ANC card and jot some information]*** | | |
| **Information on birth preparedness for the index pregnancy** | | | |
| **8. Birth preparedness**: Are you prepared on the following items for the index pregnancy? **(prompt and circle response)**   - Saving money 1. Yes 2. No - Place of delivery 1. Yes 2. No - Transportation 1. Yes 2. No - Items for the newborn babies 1. Yes 2. No - Social support (someone to assist with domestic work during postpartum period)   1. Yes 2. No | | | |

## C1-B: EXTRACTION FORM - (for *RCH-4 or ANC Card English Version)

| ****RCH-4 (Mother’s Health card during pregnancy, delivery, and postnatal periods)***  **Demographic and obstetric characteristics of pregnant woman, and ANC services received** | | | |
| --- | --- | --- | --- |
| **1.** | Date of Birth: [**dd/mm/yyyy**] /____/____/_________/ | \|____\|years (complete) | |
| **2.** | Date of last ANC visit : [**dd/mm/yyyy**] /____/____/_________/ |  | |
| **3.** | Gestation week as per last ANC visit: | \|____\|weeks | |
| **4.** | Number of index pregnancy (parity) | \|____\|pregnancies | |
| **5.** | Gestation age of pregnancy in weeks at your first ANC visit  Date [dd/mm/yyyy] | | \|_________\|weeks  **/____/____/20__** |
| **6.** | Gestation age of pregnancy in weeks at last ANC visit  Date [dd/mm/yyyy] | | \|_________\|weeks  **/____/____/20__** |
| **7.** | **Record following measurements from the ANC card: (Last measurement)** | | |
|  | Weight in kilogram | | \|_________\| kgs |
|  | Height in centimeters | | \|_________\| cms |
|  | Blood pressure | | Sys \|____\|  Dia ­­­\|_____\| |
|  | Hemoglobin level (Hb) | | Hb \|________\|g/dL |
|  | HIV tested **(circle response)** | | 1. Yes 2. No |
|  | HIV status **(circle response)** | | 1. Pos 2. Neg  3. Unknown |
|  | Syphilis tested **(circle response)** | | 1. Yes 2. No |
|  | Syphilis status **(circle response)** | | 1. Pos 2. Neg |
|  | Anti-helminth first dose **(circle response)** | | 1. Yes 2. No |
|  | Anti-helminth second dose **(circle response)** | | 1. Yes 2. No |
|  | IPTp first dose **(circle response)** | | 1. Yes 2. No |
|  | IPTp second dose **(circle response)** | | 1. Yes 2. No |
|  | Iron and folic acid supplements **(circle response)** | | 1. Yes 2. No |
|  | Tetanus immunization (first dose) **(circle response)** | | 1. Yes 2. No |
|  | Tetanus immunization (second dose) **(circle response)** | | 1. Yes 2. No |

****************************END OF RECORDING**********************************
